# Diversity and distribution of chironomids in Central European ponds

**DOI:** 10.1002/ece3.11354

**Published:** 2024-05-05

**Authors:** Marcela Sedlačková Přidalová, Ladislav Hamerlík, Milan Novikmec, Veronika Slobodníková, Marta Veselská, Peter Bitušík, Marek Svitok

**Affiliations:** ^1^ Department of Biology and General Ecology Technical University in Zvolen Zvolen Slovakia; ^2^ Department of Biology and Ecology Matej Bel University Banská Bystrica Slovakia; ^3^ Institute of Zoology Slovak Academy of Sciences Bratislava Slovakia; ^4^ Department of Forest Ecology, Faculty of Forestry and Wood Sciences Czech University of Life Sciences Prague Prague Czech Republic; ^5^ Plant Science and Biodiversity Center Slovak Academy of Sciences Bratislava Slovakia

**Keywords:** the Carpathians, Diptera, lentic waters, Nematocera, Pannonian plain

## Abstract

Ponds are common freshwater habitats in the European landscape that substantially contribute to local and regional biodiversity. Chironomids often dominate invertebrate communities in ponds but are usually disregarded in ecological studies due to relatively complicated taxonomy and identification issues. We present a comprehensive overview of the chironomid diversity in 246 ponds spanning a wide range of conditions extending from the Pannonian Plain to the Carpathians. Altogether, we recorded 225 taxa including 192 species from six subfamilies (Podonominae, Tanypodinae, Diamesinae, Prodiamesinae, Orthocladiinae and Chironominae). However, the chironomid taxa inventory is far from complete and about 16% of the total diversity of pond‐dwelling chironomids remains undetected. Chironomid alpha diversity showed a significant unimodal pattern along the elevation gradient with the highest number of taxa per pond expected around 790 m a.s.l. Gamma diversity also peaked in mid‐elevations (600–800 m), and the common chironomid taxa partitioned the 2100‐m long altitudinal gradient relatively evenly. The heterogeneity of chironomid communities among ponds measured as beta diversity was significantly higher in elevations below 800 m. Temperature and the proportion of surrounding forests significantly influenced alpha diversity of chironomid communities, while urban land cover and pond size had no significant effect. Ponds with a mean annual air temperature of approximately 4.8°C and a low proportion of surrounding forests are expected to harbour the most diverse chironomid communities. Our study showed that chironomids represent a very diverse and often exceptionally rich group of pond‐dwelling macroinvertebrates. Given the high diversity and broad range of occupied niches, chironomids should not be overlooked in pond ecology studies. On the contrary, they should be considered a potential model group.

## Graphical abstract

Chironomids represent a highly diverse and often exceptionally rich group of aquatic invertebrates that are frequently overlooked in studies of pond ecosystems. Temperature and the proportion of surrounding forests significantly influence their local diversity, whereas urban land cover and pond size show no significant effect. Given their high diversity and the broad range of occupied niches, chironomids should be considered a valuable model group in pond ecology studies.

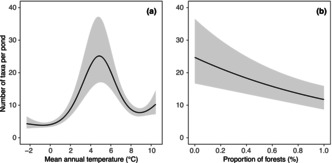

## 1 INTRODUCTION

Ponds are common freshwater habitats in the European landscape that may substantially contribute to local and regional biodiversity and serve as refugia for many rare and endangered species (Svitok et al., 2018; Williams et al., 2004). Ponds are often considered sentinels of environmental changes due to their rapid response to environmental triggers (Oertli et al., 2009). Except for their significant contribution to aquatic biodiversity (Hamerlík et al., 2014; Ilg & Oertli, 2013; Oertli et al., 2009), ponds also play an important role in catchment processes such as nutrient retention, rainfall interception, or carbon sequestration (Céréghino et al., 2014; Oertli et al., 2009). While ponds are vital in both ecological and societal contexts, freshwater research has historically focused on larger water bodies, resulting in lower priority for efforts to understand and conserve pond ecosystems (Hill et al., 2021).

Chironomidae (Diptera, Nematocera) represent a major part of benthic invertebrates in many freshwater ecosystems and often dominate invertebrate communities in ponds (Batzer & Ruhí, 2013; Fuentes‐Rodriguez et al., 2013; Medeiros & Quinlan, 2011; Novikmec et al., 2015). Nevertheless, ecological research involving chironomids typically focuses on streams and lakes while studies of ponds are rare (e.g., Campbell et al., 2009; Cantrell & McLachlan, 1982; Driver, 1977; Leeper & Taylor, 1998). Chironomids occupy a broad range of habitats and can reach high densities even in sites with unfavourable conditions, such as extremely low pH values (Rodrigues & Scharf, 2001), drying up of habitats (Frouz et al., 2003), low oxygen concentration (Brodersen et al., 2004) or high degree of pollution (Armitage et al., 1995). Some species can tolerate extreme water and air temperatures ranging from less than −20°C (Bouchard Jr et al., 2006) to almost 40°C (Hayford et al., 1995). The broad distribution of chironomids and their diverse environmental preferences make them a valuable model group for studying environmental changes in freshwater ecosystems (e.g., Céréghino et al., 2008; Eggermont & Heiri, 2012; Lindegaard, 1995; Pinder, 1986). Despite their high indicator potential, chironomids are often disregarded in ecological studies due to relatively complicated taxonomy and identification issues.

To bridge the gaps in knowledge of pond‐dwelling chironomids, we used a robust dataset from 246 Central European ponds to assess species inventory completeness (aim 1) and to investigate chironomid diversity patterns along an altitudinal gradient exceeding 2100 m (aim 2). Since altitude is rather a proxy variable than a true driver of species distributions, our specific aim was to assess the influence of climate and land use on the diversity of chironomid communities in ponds (aim 3).

Several studies (e.g., Matthews‐Bird et al., 2016; Walker et al., 1991, 1992; Walker & Mathewes, 1989) have convincingly demonstrated that climatic parameters are among the most important environmental variables that can help explain the extensive geographic distribution of chironomids. Temperature, in particular, is a critical factor that directly modulates the physiological and behavioural processes of organisms (Brown et al., 2004). According to the physiological tolerance hypothesis (Currie et al., 2004), the distribution of individual species is limited by their climatic tolerances and therefore the sum of these distributions produces patterns of diversity. This hypothesis suggests that a greater range of physiological parameter combinations can survive in warm conditions than in cold environments. Therefore, we expect that the diversity of pond‐dwelling chironomid communities will increase with the rising temperature of the environment.

Chironomids also respond sensitively to changes in land use, including urbanization, agriculture, deforestation, and subsequent nutrient enrichment (Campbell et al., 2009; Fenoy & Casas, 2015; Francis & Foster, 2001). These activities can have significant impacts on the water quality and habitat conditions. For example, urbanization can lead to the loss of natural habitats, increased impervious surface areas, and pollution from human activities, leading to degraded aquatic environments (Paul & Meyer, 2001; Thornhill et al., 2017). Owing to the specificity of the pond environment (Hamerlík et al., 2014; Richardson et al., 2022), the responses of chironomid communities to land use changes may differ from those observed in lakes and streams. In particular, we expect a sensitive response of local diversity to the changes in terrestrial land cover, given the tight linkages between the quality of the pond environment and local land use (Novikmec et al., 2016).

## 2 MATERIALS AND METHODS

### 2.1 Study sites

**FIGURE 1:**
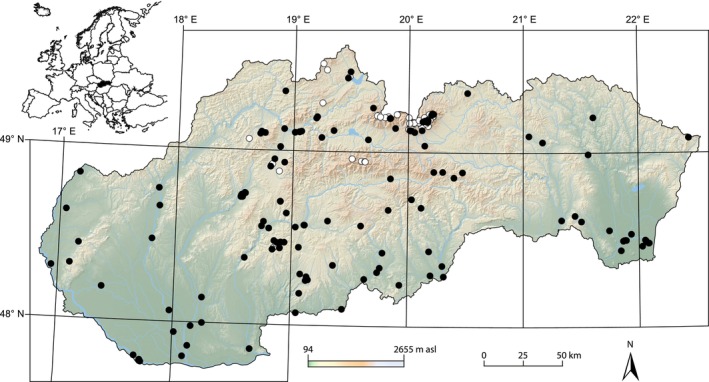
Spatial distribution of 246 ponds sampled for chironomids in Slovakia. Sites with published and unpublished data are depicted by white and black dots, respectively. For the altitudinal distribution of sampling sites, see Figure S1a.

For the purposes of this study, we considered lentic waterbodies with surface area < 2 ha and maximum depth < 8 m as ponds (see also Hamerlík et al., 2014; Oertli et al., 2000). The dataset includes published and unpublished data from 246 ponds in Slovakia (Table S1). The ponds were distributed over a wide range of geographical and ecological conditions extending from the lowlands to the alpine zone (Figure 1). The sampled ponds spanned altitudes from 97 to 2201 m (median = 736 m), surface areas from 0.0012 to 1.98 ha (0.19 ha) and depths from 0.04 to 7.5 m (0.92 m). We included temporary, semi‐permanent and permanent ponds of both natural and artificial origin. Data from repeatedly sampled sites were pooled and presented only once.

### 2.2 Field sampling and laboratory works

Most of the data used in this study (180 ponds) stem from our large‐scale field surveys of larvae and pupal exuviae (Table S1). Both the PLOCH method (Oertli et al., 2005) and semiquantitative kicking were used to sample larvae. As the first step of the PLOCH method, microhabitats were identified and their coverage was estimated according to Oertli et al. (2005). Four to eight subsamples were taken at each site (median = 7) depending on the pond area and microhabitat diversity. Subsamples were distributed among the microhabitats proportionally, with a minimum of one subsample per microhabitat. Microhabitat sampling was performed by sweeping and kicking, using a net with a rectangular frame of size 15 × 11 cm and mesh size 0.5 mm for a fixed time of 30 s. At each site, subsamples were pooled into a composite sample. When the semiquantitative kicking method was applied, each pond was sampled for a fixed time of 3 min with sampling effort proportionally distributed according to the coverage of microhabitats using a D‐shape net with a 0.5 mm mesh size.

In addition to PLOCH and kicking methods, the Chironomid Pupal Exuviae Technique – CPET (Wilson & Ruse, 2005) was employed. In CPET, floating chironomid material was collected along the shores of ponds, mostly at the leeward shore, by skimming the water surface using a hand net attached to a telescopic handle (mesh size 250 μm, frame diameter 25 cm). The sampled material was fixed in 4% formaldehyde in the field and stored in plastic bottles. In the laboratory, larvae and pupal exuviae were hand‐sorted and preserved in 75% ethanol. Afterwards, larvae and pupal exuviae were mounted in permanent slides using Berlese's mounting medium and identified using Klink and Moller Pillot (2003), Langton and Visser (2003), Orendt and Spies (2012), Andersen et al. (2013), and Bitušík and Hamerlík (2014).

### 2.3 Chironomid and environmental data

The diversity of chironomid communities was quantified at three hierarchical scales – local diversity (alpha), among‐pond variability (beta) and total diversity (gamma). Alpha diversity was defined as the number of chironomid taxa observed per pond. To calculate gamma diversity, we divided the altitudinal gradient into eleven 200‐m altitudinal belts (0–200 m, 200–400 m, etc.) and estimated the total number of chironomid taxa expected in each belt. Since the number of ponds varied widely among the belts (range = 1–52; mean = 23 ponds per belt), we estimated the expected total number of taxa for each belt using extrapolation of incidence data based on a Bernoulli product model (Colwell et al., 2012) using iNEXT library (Chao et al., 2014) in R (R Core Team, 2022). The belt from 1000 to 1200 m was excluded from the calculations as it contained only one sampling site (Figure S1a). Finally, we used Whittaker's original approach (Whittaker, 1960) and measured beta diversity (β_W_) as a ratio of gamma diversity to average alpha diversity for each altitudinal belt.

Corine Land Cover database (European Environment Agency, 2018) was used to identify land cover classes in a 500‐m buffer using the R library terra (Hijmans, 2023). The original fine classification of the classes was simplified to five categories that were sufficiently abundant in watersheds of studied ponds [mean, min–max cover]: agriculture [37, 0%–100%], forests [19, 0%–100%], grasslands [22, 0%–100%], bare rocks [13, 0%–100%] and urban areas [8, 0%–100%].

Annual air temperature [mean = 4.9, range = −2.3–10.5°C] and precipitation data [1001, 497–1601 mm] were extracted from the WorldClim 2.1 database at a 30‐arc‐sec resolution (Fick & Hijmans, 2017) providing information on the near‐current climate conditions representing averages for 1970–2000.

### 2.4 Data analysis

To assess the completeness of the taxonomic inventory (aim 1), we constructed an analytical sample‐based rarefaction curve with unconditional confidence intervals (Colwell et al., 2004) using iNEXT library (Chao et al., 2014) in R (R Core Team, 2022). We also used the asymptotic richness estimator Chao2 (Chao, 1984) to estimate the total number of pond‐dwelling chironomids, including those unobserved. The estimator was calculated using Spade (Chao & Shen, 2010).

We further explored altitudinal patterns of chironomid diversity (aim 2). Alpha diversity has been investigated using generalized additive models – GAM (Hastie & Tibshirani, 1990) implemented in the R library mgcv (Wood, 2017). GAM is a versatile semi‐parametric method that enables the fitting of a broad spectrum of response patterns. In the GAM analysis, altitude was modelled using a smooth function, while log‐transformed pond area was incorporated as a parametric function to account for possible species‐area relationships (He & Legendre, 1996). To prevent biologically less‐plausible altitudinal patterns, we constrained the level of smoothness by setting the upper limit on the degrees of freedom to five and fitted GAM with thin plate regression splines as a smoothing basis (Wood, 2003). Initially, alpha diversity was fitted using GAM with a Poisson distribution and logarithmic link function through the restricted maximum likelihood method. As the dispersion parameters of the Poisson model deviated considerably from one (φ = 4.5), we proceeded to re‐fit the data using GAM with a negative binomial distribution. In this approach, the dispersion parameter was estimated alongside the smoothing parameters to accommodate the overdispersion observed in the data. Nevertheless, spline correlograms (Bjørnstad, 2020; Bjørnstad & Falck, 2001) revealed substantial spatial autocorrelation in the model residuals. Consequently, we opted for a more complex autoregressive negative binomial GAM that incorporated an exponential spatial correlation structure (Pinheiro & Bates, 2000). The spatial covariate was derived via principal coordinates of neighbour matrices – PCNM (Borcard & Legendre, 2002) as implemented in the R library vegan (Oksanen et al., 2022). PCNM is based on the computation of the principal coordinates of a truncated pairwise geographic distance matrix among the sampling sites. We used the tenth eigenvector as a predictor in correlation structure to get autoregressive GAM with spatially independent residuals. The autoregressive GAM clearly outperformed the model assuming independent observations (ΔAIC = 868). The significance of parametric and smooth terms was assessed using Wald *t* and *F* tests, respectively (Wood, 2013).

Since altitude is a complex environmental gradient amalgamating a plethora of factors influencing biotic communities, we employed GAM with the same settings as above to discern the key climate and land use drivers of chironomid alpha diversity (aim 3). Prior to analysis, we combined climatic and land cover data in a predictor matrix and calculated pairwise correlations among variables. Air temperature was strongly correlated with the proportion of grasslands (Pearson *r* = −.69), bare rocks (*r* = −.69), agricultural landscape (*r* = .79) and precipitations (*r* = −.99). To avoid multicollinearity problems, we removed those correlated variables from the predictor matrix. Then, we fitted GAM involving smooth functions of temperature, the proportion of urban landscape and forests, along with a parametric function of log‐transformed pond area. Again, autoregressive negative binomial GAM showed better performance than the simpler models.

The graphical outputs were created using the R library ggplot2 (Wickham, 2006).

## 3 RESULTS

### 3.1 Species inventory

The checklist of pond‐dwelling chironomids in Slovakia consists of 225 taxa, with 192 of them identified at the species level. They belong to six subfamilies (Podonominae, Tanypodinae, Diamesinae, Prodiamesinae, Orthocladiinae and Chironominae) (Table S2). Chironominae was the most diverse subfamily with 104 taxa, followed by Orthocladiinae (81), Tanypodinae (29), Diamesinae (5), Prodiamesinae (2) and Podonominae (1). The most common taxa were *Procladius* (*Holotanypus*) *choreus* (60 ponds), *Synendotendipes* spp. (56) and *Corynoneura scutellata* group (55).

**FIGURE 2:**
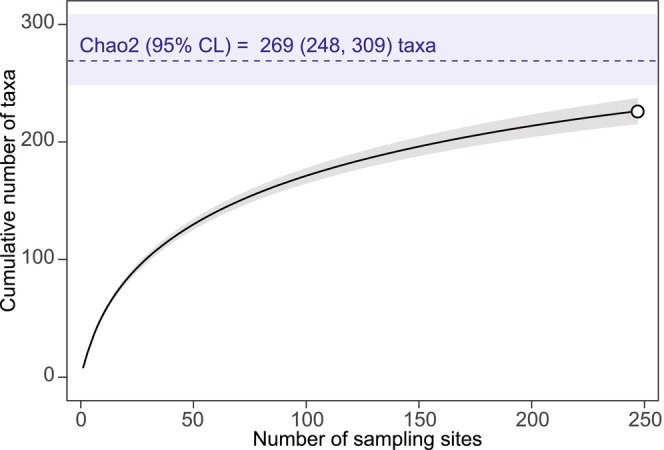
Sample‐based rarefaction curve of the taxonomic richness of pond‐dwelling chironomids in Slovakia. The shaded area around the curve indicates the 95% confidence interval. The dashed line (±95% confidence interval in blue) represents an estimate of the total number of taxa based on a non‐parametric Chao2 estimator.

However, the chironomid taxa inventory is far from complete as apparent from the rising accumulation curve which did not approach an asymptote (Figure 2). The expected total number of taxa estimated by Chao2 was 269 meaning that as many as 43 taxa remain undetected (~16% of the total number of taxa).

### 3.2 Distribution patterns

Chironomid taxa partitioned the elevation gradient relatively evenly as indicated by the distribution of their altitudinal optima (Figure S1b). The broadest altitudinal amplitude was recorded for *Paratanytarsus austriacus* (elevation range of 2103 m), *Limnophyes* spp. (2022 m) and *Metriocnemus hygropetricus* group (1992 m) (Figure S2). The majority of taxa were characteristic for lowland and upland ponds (<500 m a.s.l.), although a few taxa (e.g., *Zalutschia tatrica*, *Diamesa* spp., *Smittia* spp.) were typical for high mountain areas.

**FIGURE 3:**
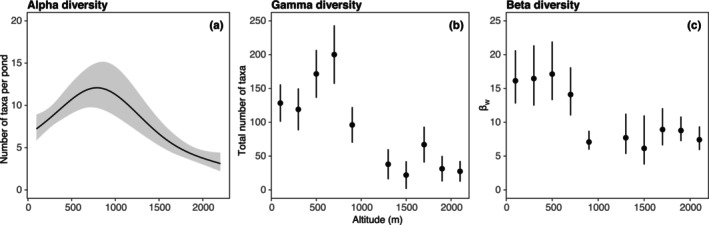
Altitudinal distribution of chironomid alpha diversity (a), gamma diversity (b) and beta diversity (c). GAM‐based prediction of alpha diversity (line) is displayed along with its 95% confidence intervals (grey band). Gamma and beta diversity (β_W_) estimates are plotted for 200‐m altitudinal belts (circles ±95% confidence error bars). Note that the 1000–1200‐m belt is missing due to a low number of sampling sites (*n* = 1).

Chironomid alpha diversity showed a clear significant unimodal pattern along the elevation gradient (effective degrees of freedom – edf = 2.7, *F* = 14.5, *p* < .0001) with the highest number of taxa per pond expected around 790 m a.s.l (Figure 3a). Conversely, pond size exhibited no significant influence (*t* = −0.67, *p* = .505).

Gamma diversity displayed a similar unimodal pattern with the highest total number of taxa expected in the altitudinal belt from 600 to 800 m (Figure 3b). In contrast, heterogeneity of chironomid communities among ponds measured by beta diversity was significantly higher in elevations below 800 m while the communities of high‐altitude ponds were more homogeneous (Figure 3c).

### 3.3 Diversity drivers

**FIGURE 4:**
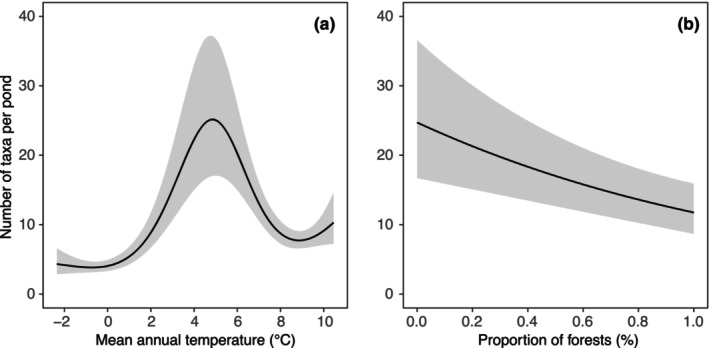
Significant relationships between chironomid alpha diversity and mean annual temperature (a) and proportion of forests in 500‐m buffer around ponds (b). GAM‐based predictions (lines) are displayed along with their 95% confidence intervals (grey bands).

The alpha diversity of chironomid communities was significantly influenced by temperature (edf = 3.8, *F* = 19.7, *p* < .0001) and the proportion of forests in the pond surroundings (edf = 1.0, *F* = 3.6, *p* < .0001) while the land cover of urban areas (edf < 0.1, *F* < 0.1, *p* = .603) and pond size (*t* = −1.1, *p* = .265) did not show a significant effect. Ponds with a mean annual air temperature of about 4.8°C and a low proportion of forests in their surroundings are expected to support the most diverse chironomid communities (Figure 4).

## 4 DISCUSSION

### 4.1 Species inventory

We summarized all available published data and complemented them with unpublished data obtained during extensive field research on pond biodiversity in Slovakia. The resulting checklist represents the first comprehensive study covering 246 ponds from the lowlands to the alpine zone and contains 225 chironomid taxa, including 192 species.

The majority of studies on the biodiversity of Chironomidae in Europe have focused on aquatic habitats other than ponds. Studies carried out in ponds are scarce and often based on a relatively small number of sites (e.g., Bazzanti et al., 2008; Leeper & Taylor, 1998) which limits drawing firm conclusions. However, the most recently published checklist of chironomids in Slovakia consists of 384 species (Bitušík & Brabec, 2009). Since its publication, several new species have been recorded (e.g., Hamerlík et al., 2015, 2019, 2022; Novikmec et al., 2013, 2015; Ščerbáková & Bitušík, 2015; Štillová et al., 2016; Štillová & Ščerbáková, 2019), and currently there are about 400 chironomid species known from Slovakia (Bitušík, unpublished data). Pond‐dwelling species comprise approximately half of all known chironomid species in Slovakia, providing indirect evidence that highlights ponds as important biodiversity hotspots. Using non‐chironomid taxa, Williams et al. (2004) compared the biodiversity of streams, rivers, lakes and ponds and reported that although all waterbody types contributed to the aquatic invertebrate diversity in the region, ponds supported considerably more species, more unique species, and more scarce species than the other habitats. Our findings corroborate the general perception of ponds as habitats with high conservation value.

Moreover, from the species accumulation curve and the Chao2 estimator, it is evident that the species inventory of pond‐dwelling chironomids in Slovakia is incomplete, with many species remaining undetected. Thus, there is a high probability of discovering dozens of other chironomid species in additional ponds. Exploring understudied habitats like wetlands, fens, peat bogs, acid mine waters, and oxbow ponds with diverse hydrological conditions may reveal new chironomid species.

### 4.2 Distribution patterns

The altitudinal distribution of alpha and gamma diversity of pond‐dwelling chironomids followed an unimodal or ‘hump‐shaped’ pattern with the highest number of taxa expected at middle elevations (600–800 m a.s.l.).

The unimodal altitudinal pattern of chironomid diversity observed in our study has also been documented in a few other chironomid studies (e.g., de Mendoza & Catalan, 2010; Nyman et al., 2005). According to de Mendoza and Catalan (2010), this unimodal pattern could be due to extreme environments at very high altitudes or favourable environmental factors at intermediate‐altitude zones. Also, Nyman et al. (2005) observed a unimodal pattern of chironomid diversity along the climatic gradient with maximum richness occurring at middle elevations (400 m a.s.l.). They argue that the peak of richness may be related to the ecotonal effect where the highest diversity occurred in the transition between the boreal coniferous forest and the arctic tundra.

In general, the unimodal altitudinal pattern of chironomid diversity can arise from several stochastic and deterministic mechanisms that often act in concert (Colwell et al., 2004). High habitat and food resource diversity, moderate climatic conditions, as well as low competition may provide favourable conditions at intermediate altitude zones (Nyman et al., 2005). In contrast to the optimal conditions, chironomid diversity usually decreases towards higher elevations. This decline in diversity can be explained by environmental forcing related to low temperature and resource availability (Eggermont & Heiri, 2012; Oliver, 1971; Pinder, 1986), historical events (Allegrucci et al., 2006; Krosch et al., 2011; McKie et al., 2005), and to the limited dispersal ability of aquatic insects in high altitudes (Ashe et al., 1987; Bitušík et al., 2017). On the other hand, such a hump‐shaped pattern may be interpreted as a result of purely stochastic processes that generate geometric constraints – the so‐called ‘mid‐domain’ effect (Colwell et al., 2004; Colwell & Hurtt, 1994; Colwell & Lees, 2000). We cannot claim that these null expectations are somehow more important than other causes (e.g., temperature, resource availability, and historical events); most likely both stochastic and multiple environmental factors drive the observed peak in chironomid species richness at medium elevations.

We have also shown that ponds in lower altitudes (<800 m) have higher beta diversity than those in higher elevations. Various mechanisms can contribute to the emergence of this pattern. Notably, habitat heterogeneity is commonly regarded as the primary driver for promoting freshwater beta diversity (Hamerlík et al., 2014; Suurkuukka et al., 2012). More variable habitats can support a wider range of assemblages, a feature reflected in higher beta diversity. Nonetheless, the heterogeneity of environmental conditions between studied ponds at lower and higher altitudes is statistically comparable, as indicated by the distance‐based test of homogeneity of multivariate dispersion (Anderson, 2006) (pseudo‐*F* = 2.8, *p* = .095). Another interpretation is the effect of environmental filtering (Poff, 1997). The harsher conditions at higher altitudes, particularly lower temperatures that limit the rate of physiological processes of ectotherms (Clarke & Gaston, 2006), can result in a reduction of the regional species pool and, consequently, the higher similarity of local communities consisting of well‐adapted species (Füreder, 2007). Moreover, the regional species pool contributing to the beta diversity may also be indirectly constrained by the smaller geographic extent of higher‐elevation belts (Romdal & Grytnes, 2007). Unfortunately, conducting a more detailed comparison of our results with other chironomid studies is challenging due to the scarcity of research on this topic. Surprisingly little is known about the altitudinal patterns of beta diversity of aquatic invertebrate communities in general. Jacobsen et al. (1997) and Castro et al. (2019) observed a positive relationship between altitude and the beta diversity of aquatic invertebrate families in Neotropic streams. While these studies identified a trend opposite to that observed in our study, the underlying mechanism behind the patterns remains the same – dispersal limitation and related distance‐decaying community similarity, leading to higher beta diversity in regions where distances among sampling sites are larger.

Considering the distribution of individual taxa, we observed that chironomids partitioned the whole elevation gradient although several taxa displayed a very broad distribution range spanning approximately 2000 m. According to Stevens (1992), altitudinal tolerance is correlated with the extent of the geographic range of each species. Species that are widely distributed are expected to occur in a larger range of altitudes in contrast to those that have limited distribution. Thus, it is not surprising that some taxa with wide geographic distribution such as *Chironomus anthracinus* group, *Corynoneura scutellata* group, *Metriocnemus hygropetricus* group (Lindegaard, 1979; Sæther, 1995; Sæther & Spies, 2013) showed a very broad altitudinal range in our study. The most frequent taxa within our study – *Procladius* (*Holotanypus*) *choreus*, *Synendotendipes* spp. and *Corynoneura scutellata* group – are also widespread throughout most of Europe (Spies & Sæther, 2004).

Nevertheless, it is not entirely clear whether the broad ecological tolerances and wide distribution of taxa relate to the eurytopic distribution of some taxa or because they consist of several cryptic species with narrower ecological valences. For example, *Paratanytarsus austriacus* and *Heterotrissocladius marcidus* are regarded as cold‐stenotherms and typically occur in high‐altitude lakes and ponds of the Alps (Boggero et al., 2006) and Tatra Mountains (Hamerlík et al., 2017; Hamerlík & Bitušík, 2009). However, we frequently found these taxa in lowland ponds, which contradicts the expected environmental preferences. The presence of various cryptic species sharing similar morphology but having distinct ecological requirements might explain these counterintuitive patterns. For instance, Ballayová et al. (2015) found 85 distinct haplotypes of *Heterotrissocladius marcidus* in the Tatra Mountains and proposed that this taxon could consist of multiple divergent and geographically separate species. Unfortunately, the cryptic diversity of chironomids is generally understudied and further research is needed to resolve ecological niche differentiation within complexes of morphologically similar species.

Typical cold stenothermal taxa such as *Zalutschia tatrica*, *Diamesa* spp. and *Smittia* spp. were restricted to high‐altitude ponds (>1500 m a.s.l.) and their optima were shifted towards higher elevations. This observation aligns with the findings of Rossaro (1991), who proposed that most cold stenothermal chironomids possess narrow temperature niches, with their temperature optima being close to their minimum tolerances. In contrast, warm‐water species seem to tolerate a broader altitudinal range. The optimum temperature for warm‐stenothermal species is near their maximum tolerance value (Rossaro, 1991), a pattern visible also in our study (Figure S2).

### 4.3 Diversity drivers

Our observations indicate that the most diverse chironomid communities in ponds are found in a mid‐range of the temperature gradient, with a mean annual air temperature of approximately 4.8°C. This unimodal diversity pattern contradicts the physiological tolerance hypothesis (Currie et al., 2004), which posits an increase in diversity with temperature. The other studies of chironomid alpha diversity along temperature gradients report idiosyncratic trends encompassing positive relationships (de Mendoza et al., 2023; Engels et al., 2019; Hamerlík et al., 2017), unimodal patterns (Engels et al., 2019), negative relationships (Hamerlík et al., 2017) and non‐significant responses (de Mendoza et al., 2023). This is consistent with the results of a meta‐analysis showing no universal response of local diversity to temperature and a large taxonomic and geographic specificity in these relationships (Hawkins et al., 2007).

The non‐linear response of lentic chironomid alpha diversity to temperature was also observed by Engels et al. (2019). Using a large lake dataset from the Northern Hemisphere, they showed a steep increase in diversity as July air temperatures increased between 2.5 and 14°C. However, in some areas, they noticed a subsequent stabilization or decline in diversity when the temperature exceeded 14°C. Due to a tight relationship between the annual and July temperatures in our dataset (*T*_July_ = 8.85 + 0.027 × *T*_annual_, *F* = 7464, *p* < .0001, *r* ^2^ = .996), we can easily see that the annual temperature of 4.8°C corresponds to the July temperature of 14.3°C, which closely aligns a diversity peak observed by Engels et al. (2019). The peak of richness may be related to conditions in a mid‐range of the temperature gradient that are suitable for warm and cold stenotherms, as well as eurytherms. In the mid‐temperature gradient is potential for species overlap, thereby enhancing the overall observed diversity (Nyman et al., 2005). Concerning the colder end of the temperature gradient, local chironomid diversity may be constrained by species physiological limitations and a paucity of high‐quality food resources (Eggermont & Heiri, 2012; Engels et al., 2019; Nyman et al., 2005). On the other hand, a declining diversity pattern with increasing air temperatures may result from reduced oxygen availability, elimination of macrophytes or changes in pH, all of which can have adverse effects on chironomid communities (e.g., Brodersen & Quinlan, 2006; Eggermont & Heiri, 2012; Engels et al., 2019).

The quality of the aquatic environment in ponds is directly influenced by land use in their surroundings (Novikmec et al., 2016) and chironomid communities respond sensitively to land use changes in pond catchments (Campbell et al., 2009; Francis & Foster, 2001). In the studied system, we observed a negative relationship between chironomid diversity and the proportion of forests in the surroundings of ponds. Because of the tight aquatic‐terrestrial interface of ponds, a high forest cover alone can lead to increased nutrient concentrations through direct leaching from fallen leaf litter (Biggs et al., 1994; Thornhill et al., 2017). Moreover, high canopy cover is often associated with a decrease in aquatic plant abundance, algal diversity, water temperature, and diurnal dissolved oxygen (Plenzler & Michaels, 2015). All these changes can directly influence pond‐dwelling invertebrates and, similar to our results, the high proportion of forests around ponds has been reported to reduce pond macroinvertebrate diversity, including chironomids (Batzer et al., 2004; Bischof et al., 2013; Plenzler & Michaels, 2015; Thornhill et al., 2017).

Finally, we found that pond area had no significant effect on the alpha diversity of chironomid communities. This contradicts the theory of island biogeography (MacArthur & Wilson, 1967) assuming that the number of species increases with the habitat size. Despite habitat size being regarded as a critical factor in shaping species richness patterns, various studies have reported weak or no relationship between diversity and pond area (e.g., Hamerlík et al., 2014; Martínez‐Sanz et al., 2012; Oertli et al., 2002). A meta‐analysis by Drakare et al. (2006) revealed that many habitat characteristics may have a profound effect on the species‐area relationships. The influence of size on species diversity in ponds is likely overridden by other factors or stochastic events, which can have a greater impact on ponds compared to larger, more stable water bodies.

In conclusion, we found that Central European ponds represent core habitats for aquatic chironomid species, with almost half of all known species of the family inhabiting small, shallow lentic water bodies. The combination of high diversity and a wide range of ecological niches, along with a robust response to temperature and land‐use drivers, make chironomids a promising model group of aquatic invertebrates that should not be overlooked by researchers focusing on pond ecosystems.

## AUTHOR CONTRIBUTIONS

**Marcela Sedlačková Přidalová:** Conceptualization (supporting); data curation (lead); investigation (equal); resources (equal); writing – original draft (equal); writing – review and editing (equal). **Ladislav Hamerlík:** Funding acquisition (equal); investigation (equal); resources (equal); writing – review and editing (equal). **Milan Novikmec:** Funding acquisition (equal); investigation (equal); project administration (lead); writing – review and editing (equal). **Veronika Slobodníková:** Investigation (equal); resources (equal); writing – review and editing (equal). **Marta Veselská:** Investigation (equal); resources (equal); writing – review and editing (supporting). **Peter Bitušík:** Investigation (equal); resources (equal); writing – review and editing (supporting). **Marek Svitok:** Conceptualization (lead); data curation (supporting); formal analysis (lead); funding acquisition (equal); investigation (equal); methodology (lead); project administration (equal); supervision (lead); visualization (lead); writing – original draft (equal); writing – review and editing (equal).

## FUNDING INFORMATION

The study was supported by the Slovak Research and Development Agency under contract No. APVV‐16‐0236, the scientific grant agency VEGA (projects No. 1/0400/21 and 2/0044/22) and the Operational Programme Integrated Infrastructure funded by the European Regional Development Fund (ITMS 313011T721).

## CONFLICT OF INTEREST STATEMENT

All authors declare no conflict of interest.

## Supporting information


Data S1:

## Data Availability

The data that supports the findings of this study are available in the supplementary material of this article.
