# Supplementary material for: Diversity and distribution of chironomids in Central European ponds
Source: Ecol Evol. 2024 May 5;14(5):e11354. doi: 10.1002/ece3.11354 (PMC11070637; doi:10.1002/ece3.11354)
Supplement: Supplementary file 1 — Data S1: [file ECE3-14-e11354-s001.docx]

**Supporting information**

**
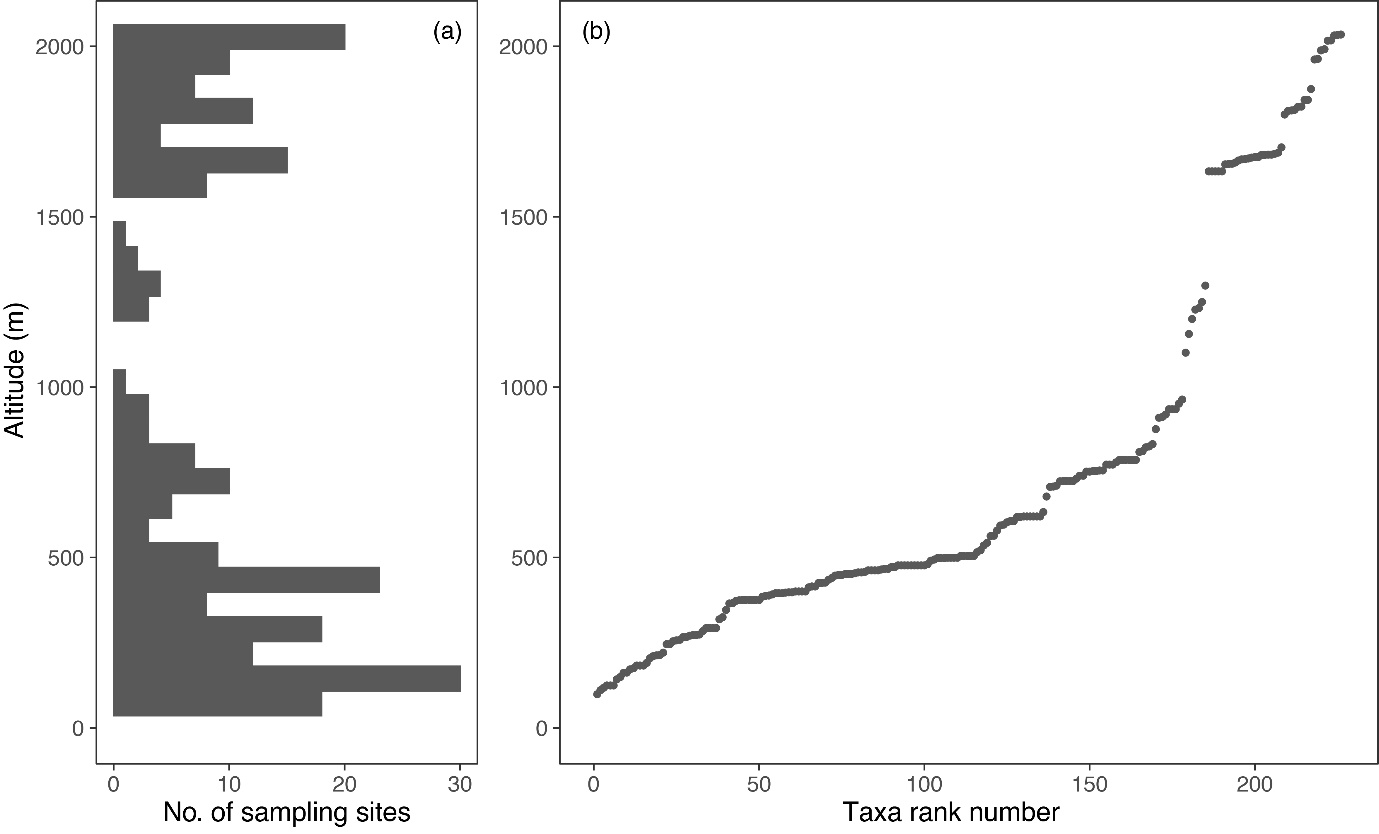
**

**Figure S1** Distribution of sampled ponds (b) and position of altitudinal optima of chironomid taxa (a). The optima are represented by median elevations of taxa distributions in studied ponds. For numeric details see Table S2.

**
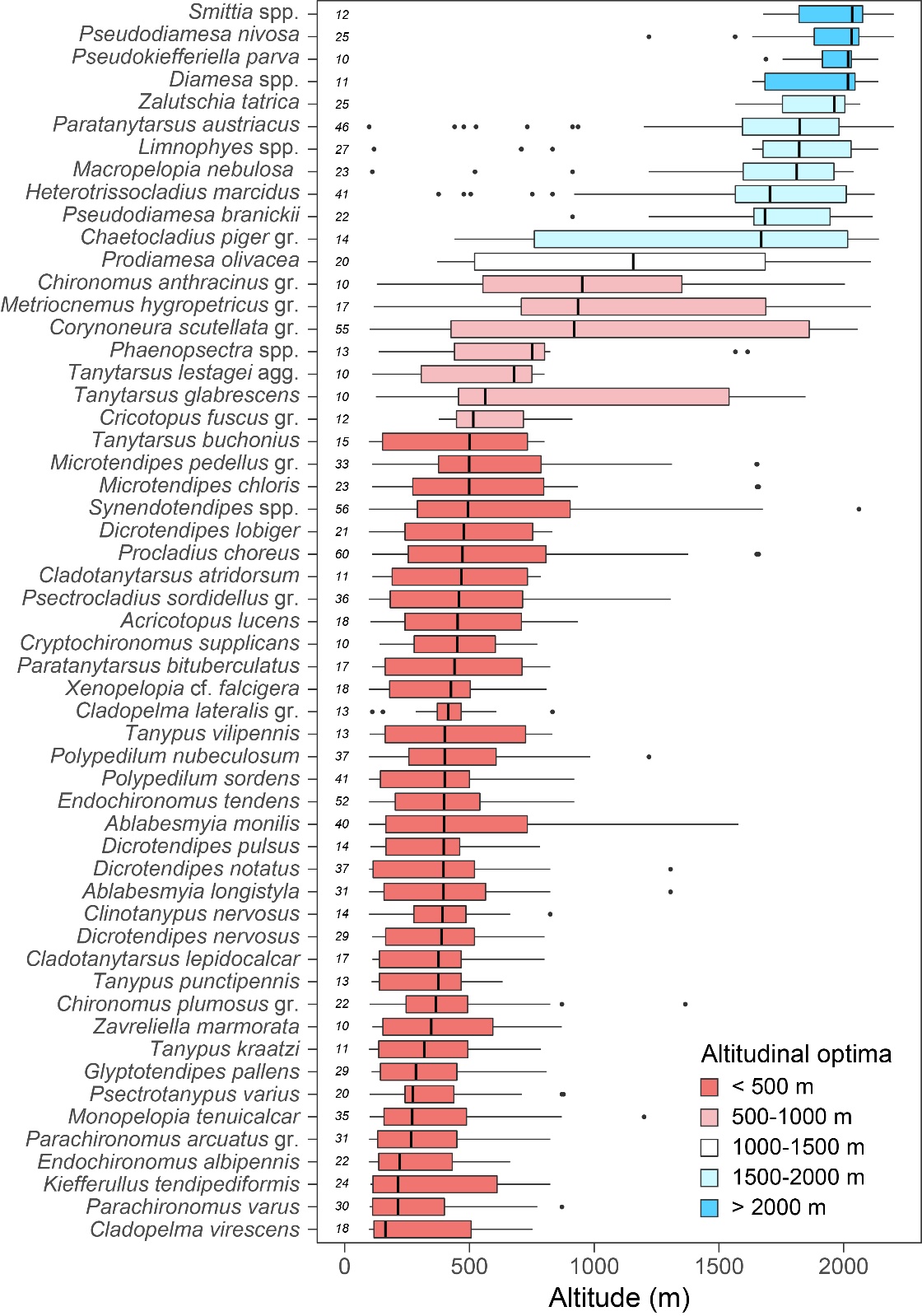
**

**Fig. S2** Altitudinal distribution of the chironomid taxa in sampled ponds. Taxa are ordered according to the position of their altitudinal optima (median of distribution). The number of sampling sites for each taxon is given in italics. Note that only frequently occurring taxa (≥ 10 sites) are displayed.

**Table S1** Overview of sampled ponds involved in the study. For more details see Material and Methods.

| **Region** | **Larvae collection method** | **Exuviae collection method** | **No. of ponds** | **Sampling date** | **Reference** |
| --- | --- | --- | --- | --- | --- |
| whole Slovakia | PLOCH | hand net | 84 | 2012‑2013 | unpublished data |
| Central Slovakia | PLOCH | ‑ | 23 | 2018‑2019 | unpublished data |
| Central Slovakia | ‑ | hand net | 5 | 2014‑2019 | unpublished data |
| Latorica River floodplain | Semiquantitative kicking | ‑ | 13 | 2017 | unpublished data |
| Danube River floodplain | Semiquantitative kicking | ‑ | 6 | 2017 | unpublished data |
| Košské mokrade Wetlands | Semiquantitative kicking | hand net | 12 | 2006‑2007 | unpublished data |
| Tatra Mts. | Semiquantitative kicking | ‑ | 37 | 2013‑2016 | unpublished data |
| Tatra Mts. | Semiquantitative kicking | ‑ | 47 | 2000‑2013 | Novikmec *et al*., 2015; Hamerlík *et al*., 2017 |
| Štiavnické vrchy Mts. | ‑ | hand net | 5 | 2005 | Bitušík *et al*., 2007 |
| Southern part of Central Slovakia | ‑ | hand net | 2 | 1987 | Bitušík, 1988 |
| Low Tatra Mts. | Semiquantitative kicking | hand net | 4 | 1993‑1994 | Bitušík & Koppová, 1997 |
| Wetlands in Northern Slovakia | ‑ | hand net | 8 | 1996‑1997 | Bitušík & Turanová, 1997, Bitušík, 1998 |

**References**

Bitušík, P. (1988). *Pakomáre (Diptera: Chironomidae) vybraných vodných biotopov v povodí Ipľa*, pp. 173-190. In: Prehľad odborných výsledkov XXIII.TOP (Plachtinská dolina, 1987), ONV-OK Veľký Krtíš, SZOPK UV Bratislava.

Bitušík, P. & Koppová, K. (1997). Macrozoobenthos of the glacial lakes in the Low Tatras (West Carpathians): Aquatic insects. *Biologia,* **52**, 227–232.

Bitušík, P., Turanová, M. (1997). Letný aspekt výskytu vodného hmyzu chráneného prírodného náleziska Jazierko pri Jazernici. *Entomofauna carpatica*, **9**, 76–78.

Bitušík, P. (1998). K poznaniu pakomárov (Diptera: Chironomidae) PR Šujské rašelinisko. *Ochrana prírody*, **16**, 131–136.

Bitušík, P., Svitok, M. & Bačík, J. (2007). Chironomids (Diptera: Chironomidae) of man-made reservoirs in the Banská Štiavnica mining region (Slovakia). *Acta Zoologica Universitatis Comenianae*, **47**, 115–126.

Hamerlík, L., Svitok, M., Novikmec, M., Veselská, M. & Bitušík, P. (2017). Weak altitudinal pattern of overall chironomid richness is a result of contrasting trends of subfamilies in high-altitude ponds. *Hydrobiologia,* **793**, 67–81.

Novikmec, M., Veselská, M., Bitušík, P., Hamerlík, L., Matúšová, Z., Reduciendo Klementová, B. & Svitok, M. (2015). Checklist of benthic macroinvertebrates of high altitude ponds of the Tatra Mountains (Central Europe) with new records of two species for Slovakia. *Check List*, **11**, 1–12.

**Table S2** Checklist of 225 chironomid taxa recorded in ponds in Slovakia. The table shows the number of ponds where the species/taxon was recorded (n), median, minimum and maximum altitude of the records.

| **Chironomid taxa** | **n** | **median (min–max)** |
| --- | --- | --- |
| **Podonominae** |  |  |
| *Lasiodiamesa* sp. | 1 | 1654 (1654–1654) |
| **Tanypodinae** |  |  |
| *Ablabesmyia* cf. *phatta* (Egger, 1864) | 2 | 257 (255–259) |
| *Ablabesmyia longistyla* (Fittkau, 1962) | 31 | 396 (98–1306) |
| *Ablabesmyia monilis* (Linnaeus, 1758) | 40 | 399 (97–1577) |
| *Apsectrotanypus trifascipennis* (Zetterstedt, 1838) | 7 | 505 (396–833) |
| *Clinotanypus nervosus* (Meigen, 1818) | 14 | 392 (98–824) |
| *Conchapelopia* spp. | 8 | 413 (139–772) |
| *Derotanypus* cf. *sibiricus* (Kruglova & Chernovskii, 1940) | 2 | 1875 (1717–2032) |
| *Guttipelopia guttipennis* (van der Wulp, 1861) | 5 | 293 (183–621) |
| *Labrundinia longipalpis* (Goetghebuer, 1921) | 3 | 396 (172–401) |
| *Larsia curticalcar* (Kieffer, 1918) | 2 | 491 (477–505) |
| *Macropelopia nebulosa* (Meigen, 1804) | 23 | 1812 (111–2040) |
| *Macropelopia notata* (Meigen, 1818) | 1 | 505 (505–505) |
| *Monopelopia tenuicalcar* (Kieffer, 1918) | 35 | 271 (99–1200) |
| *Natarsia* sp. | 2 | 427 (388–465) |
| *Paramerina cingulata* (Walker, 1856) | 4 | 389 (242–477) |
| *Procladius* (*Holotanypus*) *choreus* (Meigen, 1804) | 60 | 472 (110–1659) |
| *Procladius* (*Holotanypus*) cf. *sagittalis* (Kieffer, 1909) | 3 | 274 (255–477) |
| *Procladius* (*Holotanypus*) *signatus* (Zetterstedt, 1850) | 6 | 499 (111–1306) |
| *Procladius* (*Psilotanypus*) *rufovittatus* (van der Wulp, 1875) | 2 | 463 (458–467) |
| *Psectrotanypus varius* (Fabricius, 1787) | 20 | 273 (100–877) |
| *Tanypus kraatzi* (Kieffer, 1912) | 11 | 319 (98–787) |
| *Tanypus punctipennis* (Meigen, 1818) | 13 | 375 (108–633) |
| *Tanypus vilipennis* (Kieffer, 1918) | 13 | 401 (99–833) |
| *Telmatopelopia nemorum* (Goetghebuer, 1921) | 1 | 621 (621–621) |
| *Trissopelopia flavida* (Kieffer, 1923) | 3 | 913 (505–935) |
| *Xenopelopia* cf. *falcigera* (Kieffer, 1911) | 18 | 426 (98–810) |
| *Zavrelimyia barbatipes* (Kieffer, 1911) | 4 | 500 (440–935) |
| *Zavrelimyia melanura* (Meigen, 1804) | 2 | 597 (440–754) |
| *Zavrelimyia punctatissima* (Goetghebuer, 1934) | 3 | 833 (440–913) |
| **Diamesinae** |  |  |
| *Diamesa* spp. | 11 | 2017 (1633–2139) |
| *Potthastia gaedii* (Meigen, 1838) | 1 | 376 (376–376) |
| *Pseudodiamesa branickii* (Nowicki, 1873) | 22 | 1685 (913–2117) |
| *Pseudodiamesa nivosa* (Goetghebuer, 1928) | 25 | 2032 (1219–2201) |
| *Pseudokiefferiella parva* (Edwards, 1932) | 10 | 2018 (1688–2139) |
| **Prodiamesinae** |  |  |
| *Prodiamesa olivacea* (Meigen, 1818) | 20 | 1156 (371–2109) |
| *Odontomesa fulva* (Kieffer, 1919) | 1 | 477 (477–477) |
| **Orthocladiinae** |  |  |
| *Acricotopus lucens* (Zetterstedt, 1850) | 18 | 453 (104–935) |
| *Brillia bifida* (Kieffer, 1909) | 5 | 935 (520–1688) |
| *Bryophaenocladius/Gymnometriocnemus* spp. | 8 | 1964 (1633–2067) |
| *Chaetocladius* (*Chaetocladius*) *melaleucus* (Meigen, 1818) | 1 | 935 (935–935) |
| *Chaetocladius* (*Chaetocladius*) *acuticornis* group | 2 | 1676 (1674–1677) |
| *Chaetocladius* (*Chaetocladius*) *piger* group | 14 | 1670 (440–2141) |
| *Corynoneura lobata* (Edwards, 1924) | 9 | 1665 (101–1688) |
| *Corynoneura* Pe1 (Langton, 1991) | 1 | 754 (754–754) |
| *Corynoneura* Pe4 (Langton, 1991) | 7 | 787 (725–1200) |
| *Corynoneura scutellata* group | 55 | 920 (99–2056) |
| *Cricotopus* (*Cricotopus*) *albiforceps* (Kieffer, 1916) | 2 | 813 (801–824) |
| *Cricotopus* (*Cricotopus*) *bicinctus* (Meigen, 1818) | 4 | 463 (449–505) |
| *Cricotopus* (*Cricotopus*) cf. *cylindraceus* (Kieffer, 1908) | 2 | 466 (98–833) |
| *Cricotopus* (*Cricotopus*) *festivellus* (Kieffer, 1906) | 2 | 1232 (787–1677) |
| *Cricotopus* (*Cricotopus*) *similis* (Goetghebuer 1921) | 1 | 773 (773–773) |
| *Cricotopus* (*Cricotopus*) *tibialis* (Meigen, 1804) | 3 | 773 (440–1661) |
| *Cricotopus* (*Cricotopus*) *patens* (Hirvenoia, 1973)*/flavocinctus* (Kieffer, 1924) | 1 | 183 (183–183) |
| *Cricotopus* (*Cricotopus*) *fuscus* group | 12 | 516 (376–913) |
| *Cricotopus* (*Cricotopus*) *tremulus* group | 9 | 1843 (98–2018) |
| *Cricotopus* (*Isocladius*) *intersectus* (Staeger, 1839) | 5 | 162 (108–724) |
| *Cricotopus* (*Isocladius*) cf. *laricomalis* (Edwards, 1932) | 1 | 787 (787–787) |
| *Cricotopus* (*Isocladius*) *ornatus* (Meigen, 1818) | 7 | 259 (136–526) |
| *Cricotopus* (*Isocladius*) *reversus* (Hirvenoja, 1973) | 7 | 143 (110–1843) |
| *Cricotopus* (*Isocladius*) *tricintus* (Meigen, 1818) | 6 | 544 (110–810) |
| *Cricotopus* (*Isocladius*) *trifasciatus* (Meigen, 1810) | 6 | 472 (98–1200) |
| *Cricotopus* (*Isocladius*) Pe1 (Langton, 1991) | 1 | 477 (477–477) |
| *Cricotopus* (*Isocladius*) Pe5 (Langton, 1991) | 1 | 740 (740–740) |
| *Cricotopus* (*Paratrichocladius*) *rufiventris* (Meigen, 1830) | 1 | 773 (773–773) |
| *Cricotopus* (*Paratrichocladius*) *skirwithensis* (Edwards, 1929) | 2 | 1669 (1633–1704) |
| *Diplocladius cultriger* (Kieffer, 1908) | 2 | 1228 (752–1704) |
| *Eukiefferiella brevicalcar* (Kieffer, 1911) | 2 | 1655 (1633–1677) |
| *Eukiefferiella claripennis* (Lundbeck, 1898) | 2 | 1676 (1674–1677) |
| *Eukiefferiella coerulescens* (Kieffer, 1926) | 5 | 1674 (1661–1688) |
| *Eukiefferiella minor* (Edwards, 1929)/ *fittkaui* (Lehmann, 1972) | 1 | 877 (877–877) |
| *Eukiefferiella tirolensis* (Goetghebuer, 1938) | 1 | 1688 (1688–1688) |
| *Heleniella serratosioi* (Ringe, 1976) | 1 | 1633 (1633–1633) |
| *Heterotrissocladius marcidus* (Walker, 1856) | 41 | 1704 (376–2124) |
| *Hydrobaenus conformis* (Holmgren, 1869) | 1 | 1988 (1988–1988) |
| *Hydrobaenus lugubris* group | 6 | 111 (109–139) |
| *Hydrobaenus pilipes* group | 1 | 172 (172–172) |
| *Krenosmittia boreoalpina* (Goetghebuer, 1944) | 4 | 1671 (1633–1688) |
| *Limnophyes* spp. | 27 | 1822 (117–2139) |
| *Metriocnemus hygropetricus* group | 17 | 935 (117–2109) |
| *Nanocladius* (*Nanocladius*) *rectinervis* (Kieffer, 1911) | 1 | 621 (621–621) |
| *Nanocladius* (*Nanocladius*) *balticus* (Palmen, 1959) | 1 | 500 (500–500) |
| *Nanocladius* (*Nanocladius*) *dichromus* (Kieffer, 1906) | 3 | 500 (449–724) |
| *Orthocladius* (*Eudactylocladius*) *fuscimanus* (Kieffer, 1908) | 2 | 1800 (1633–1967) |
| *Orthocladius* (*Eudactylocladius*) *olivaceus* (Kieffer, 1911) | 1 | 1633 (1633–1633) |
| *Orthocladius* (*Euorthocladius*) *ashei* (Soponis, 1990) | 2 | 756 (725–787) |
| *Orthocladius* (*Mesorthocladius*) *frigidus* (Zetterstedt, 1838) | 4 | 1250 (913–1633) |
| *Orthocladius* (*Orthocladius*) cf. *wetterensis* (Brundin, 1956) | 2 | 367 (139–594) |
| *Orthocladius* (*Orthocladius*) *oblidens* (Walker 1856) / *rhyacobius* (Kieffer, 1991) | 1 | 454 (454–454) |
| *Orthocladius* (*Symposiocladius*) *holsatus* (Goetghebuer, 1937) | 5 | 619 (454–935) |
| *Paracladius conversus* (Walker, 1856) | 3 | 477 (376–773) |
| *Paracricotopus niger* (Kieffer, 1913) | 1 | 505 (505–505) |
| *Parakiefferiella coronata* (Edwards, 1929) | 1 | 477 (477–477) |
| *Paralimnophyes* sp. | 1 | 99 (99–99) |
| *Parametriocnemus* spp. | 5 | 1810 (1661–1817) |
| *Parorthocladius nudipennis* (Kieffer, 1908) | 3 | 1681 (1633–1704) |
| *Psectrocladius* (*Allopsectrocladius*) *flavus* (Johannsen, 1905) | 1 | 273 (273–273) |
| *Psectrocladius* (*Allopsectrocladius*) *obvius* (Walker, 1856) | 8 | 708 (197–913) |
| *Psectrocladius* (*Allopsectrocladius*) *platypus* (Edwards, 1929) | 1 | 505 (505–505) |
| *Psectrocladius* (*Psectrocladius*) *limbatellus* (Holmgren, 1869) | 5 | 267 (114–913) |
| *Psectrocladius* (*Psectrocladius*) *octomaculatus* (Wuelker, 1956) | 7 | 1659 (109–1843) |
| *Psectrocladius* (*Psectrocladius*) *oligosetus* (Wülker, 1956) | 2 | 911 (621–1200) |
| *Psectrocladius* (*Psectrocladius*) *psilopterus* (Kieffer, 1906) | 7 | 183 (110–752) |
| *Psectrocladius* (*Psectrocladius*) *schlienzi* (Wülker, 1956) | 2 | 385 (293–477) |
| *Psectrocladius* (*Psectrocladius*) *sordidellus* group | 36 | 458 (97–1306) |
| *Psectrocladius* (*Psectrocladius*) *barbimanus* (Edwards, 1929) | 1 | 255 (255–255) |
| *Pseudosmittia* spp. | 9 | 1843 (256–2059) |
| *Rheocricotopus* (*Psilocricotopus*) *chalybeatus* (Edwards, 1929) | 3 | 449 (143–725) |
| *Rheocricotopus* (*Rheocricotopus*) *fuscipes* (Kieffer, 1909) | 2 | 1102 (526–1677) |
| *Rheocricotopus* (*Rheocricotopus*) *reduncus* Sæther & Schnell, 1988 | 2 | 1683 (1677–1688) |
| *Smittia* spp. | 12 | 2034 (1677–2201) |
| *Stilocladius montanus* (Rossaro, 1979) | 5 | 1681 (1565–1704) |
| *Thienemaniella* Pe 1b (Langton, 1991) | 1 | 1633 (1633–1633) |
| *Tokunagaia rectangularis* (Goetghebuer, 1940) | 2 | 1992 (1967–2017) |
| *Tokunagaia tonollii* (Rossaro, 1983) | 1 | 1633 (1633–1633) |
| *Tvetenia bavarica* (Goetghebuer 1934) | 4 | 1683 (1219–2124) |
| *Tvetenia calvescens* (Edwards, 1929) | 1 | 1633 (1633–1633) |
| *Zalutschia tatrica* (Pagast, 1935) | 25 | 1962 (1566–2067) |
| **Chironominae** |  |  |
| **Chironomini** |  |  |
| *Benthalia carbonaria* (Meigen, 1804) | 6 | 426 (114–772) |
| *Chironomus (Chironomus) anthracinus* group | 10 | 952 (129–2005) |
| *Chironomus (Chironomus) plumosus* group | 22 | 366 (100–1365) |
| *Chironomus (Lobochironomus) montuosus* (Ryser, Wulker & Scholl 1985) | *7* | 1814 (1806–1847) |
| *Cladopelma laccophila* group | *4* | 191 (125–1597) |
| *Cladopelma lateralis* group | *13* | 415 (111–833) |
| *Cladopelma virescens* (Meigen, 1818) | *18* | 163 (98–752) |
| *Cladopelma viridulum* (Linnaeus, 1767) | *1* | 183 (183–183) |
| *Cryptochironomus* cf. *defectus* (Kieffer, 1913) | 1 | 246 (246–246) |
| *Cryptochironomus obreptans* (Walker, 1856) | 2 | 176 (98–253) |
| *Cryptochironomus psittacinus* (Meigen, 1830) | 1 | 594 (594–594) |
| *Cryptochironomus supplicans* (Meigen, 1830) | 10 | 452 (139–772) |
| *Cryptotendipes holsatus* (Lenz, 1959) | 2 | 294 (110–477) |
| *Dicrotendipes lobiger* (Kieffer, 1921) | 21 | 477 (98–833) |
| *Dicrotendipes modestus* (Say, 1823) | 4 | 205 (98–300) |
| *Dicrotendipes nervosus* (Staeger, 1839) | 29 | 388 (110–801) |
| *Dicrotendipes notatus* (Meigen, 1818) | 37 | 396 (98–1306) |
| *Dicrotendipes pulsus* (Walker, 1856) | 14 | 397 (104–783) |
| *Dicrotendipes septemmaculatus* (Becker, 1908) | 1 | 376 (376–376) |
| *Dicrotendipes tritomus* (Kieffer, 1916) | 1 | 787 (787–787) |
| *Endochironomus albipennis* (Meigen, 1830) | 22 | 221 (98–663) |
| *Endochironomus tendens* (Fabricius, 1775) | 52 | 399 (98–920) |
| *Fleuria lacustris* (Kieffer, 1924) | 2 | 435 (98–772) |
| *Glyptotendipes* (*Caulochironomus*) *foliicola* (Contreras & Lichtenberg, 1997) | 4 | 452 (114–787) |
| *Glyptotendipes* (*Glyptotendipes*) *barbipes* (Staeger, 1839) | 8 | 211 (109–607) |
| *Glyptotendipes* (*Glyptotendipes*) *cauliginellus* (Kieffer 1913) | 5 | 619 (458–787) |
| *Glyptotendipes* (*Glyptotendipes*) *pallens* (Meigen, 1804) | 29 | 285 (108–810) |
| *Glyptotendipes* (*Glyptotendipes*) *paripes* (Edwards, 1929) | 7 | 457 (111–1200) |
| *Glyptotendipes* (*Heynotendipes*) *signatus* (Kieffer, 1909) | 7 | 376 (98–724) |
| *Harnischia curtilamellata* (Malloch, 1915) | 2 | 448 (143–752) |
| *Kiefferullus tendipediformis* (Goetghebuer, 1921) | 24 | 214 (104–824) |
| *Lauterborniella agrayloides* (Kieffer, 1911) | 1 | 621 (621–621) |
| *Microchironomus tener* (Kieffer, 1918) | 2 | 118 (111–125) |
| *Microtendipes chloris* (Meigen, 1818) | 23 | 499 (110–1659) |
| *Microtendipes pedellus* group | 33 | 499 (110–1653) |
| *Omisus caledonicus* (Edwards, 1932) | 1 | 621 (621–621) |
| *Pagastiella orophila* (Edwards, 1929) | 2 | 1656 (1653–1659) |
| *Paracladopelma camptolabis* (Kieffer, 1913) | 1 | 477 (477–477) |
| *Parachironomus varus* (Goetghebuer, 1921) | 30 | 213 (99–870) |
| *Parachironomus arcuatus* group | 31 | 267 (98–824) |
| *Parachironomus vitiosus* group | 5 | 376 (110–621) |
| *Parachironomus* Pe3 (Langton, 1991) | 1 | 125 (125–125) |
| *Paralauterborniella nigrohalteralis* (Malloch, 1915) | 2 | 481 (209–752) |
| *Paratendipes albimanus* (Meigen, 1818) | 6 | 374 (110–526) |
| *Phaenopsectra* *flavipes* (Meigen, 1818) | 13 | 752 (136–1615) |
| *Polypedilum* (*Polypedilum*) *arundineti* (Goetghebuer, 1921) | 3 | 621 (477–877) |
| *Polypedilum* (*Polypedilum*) *nubeculosum* (Meigen, 1804) | 37 | 401 (98–1219) |
| *Polypedilum* (*Polypedilum*) *nubifer* (Skuse, 1889) | 3 | 401 (285–451) |
| *Polypedilum* (*Pentapedilum*) *sordens* (van der Wulp, 1875) | 41 | 401 (98–920) |
| *Polypedilum* (*Pentapedilum*) *uncinatum* agg. | 7 | 810 (650–983) |
| *Polypedilum* (*Uresipedilum*) *convictum* (Walker, 1856) | 2 | 780 (253–1306) |
| *Polypedilum* (*Uresipedilum*) *cultellatum* (Goetghebuer, 1931) | 6 | 150 (111–783) |
| *Polypedilum* (*Polypedilum*) *laetum* (Meigen, 1818) | 1 | 246 (246–246) |
| *Sergentia coracina* (Zetterstedt, 1850) | 2 | 756 (725–787) |
| *Stenochironomus* sp. | 1 | 376 (376–376) |
| *Stictochironomus pictulus* (Meigen, 1830) | 2 | 634 (467–801) |
| *Synendotendipes* spp. | 56 | 495 (98–2061) |
| *Xenochironomus xenolabis* (Kieffer, 1916) | 1 | 293 (293–293) |
| *Zavreliella marmorata* (van der Wulp, 1859) | 10 | 347 (111–870) |
| **Pseudochironomini** |  |  |
| *Pseudochironomus prasinatus* (Staeger, 1839) | 3 | 725 (594–787) |
| **Tanytarsini** |  |  |
| *Cladotanytarsus* (*Cladotanytarsus*) *atridorsum* (Kieffer, 1924) | 11 | 467 (111–787) |
| *Cladotanytarsus* (*Cladotanytarsus*) *lepidocalcar* (Kruger, 1938) | 17 | 376 (111–801) |
| *Cladotanytarsus* (*Cladotanytarsus*) *mancus* (Walker, 1856) | 5 | 724 (136–801) |
| *Cladotanytarsus* (*Cladotanytarsus*) *molestus* (Hirvenoja, 1962) | 1 | 505 (505–505) |
| *Cladotanytarsus* (*Cladotanytarsus*) *vanderwulpi* (Edwards, 1929) | 1 | 752 (752–752) |
| *Micropsectra atrofasciata* (Kieffer, 1911) | 6 | 1298 (505–1688) |
| *Micropsectra contracta* (Reiss, 1965) | 2 | 564 (376–752) |
| *Micropsectra lindrothi* (Goetghebuer, 1931) | 6 | 709 (454–1219) |
| *Micropsectra notescens* (Walker, 1856) | 7 | 522 (440–1681) |
| *Micropsectra radialis* (Goetghebuer, 1939) | 2 | 2035 (2018–2051) |
| *Micropsectra roseiventris* (Kieffer, 1909) | 2 | 956 (711–1200) |
| *Paratanytarsus austriacus* (Kieffer, 1924) | 46 | 1823 (98–2201) |
| *Paratanytarsus bituberculatus* (Edwards, 1929) | 17 | 440 (110–824) |
| *Paratanytarsus brevicalcar* (Kieffer, 1909) | 1 | 621 (621–621) |
| *Paratanytarsus dissimilis* (Johannsen, 1905) | 3 | 457 (109–810) |
| *Paratanytarsus grimmii* (Schneider, 1885) | 5 | 415 (139–740) |
| *Paratanytarsus intricatus* (Goetghebuer, 1921) | 2 | 463 (458–467) |
| *Paratanytarsus laccophilus* (Edwards, 1929) | 4 | 604 (458–787) |
| *Paratanytarsus laetipes* (Zetterstedt, 1850) | 5 | 725 (162–801) |
| *Paratanytarsus lauterborni* (Kieffer, 1909) | 2 | 326 (197–454) |
| *Paratanytarsus penicillatus* (Goetghebuer, 1928) | 2 | 964 (621–1306) |
| *Paratanytarsus tenellulus* (Goetghebuer, 1921) | 3 | 787 (139–810) |
| *Rheotanytarsus muscicola* (Thienemann, 1929) | 1 | 449 (449–449) |
| *Stempellina bausei* (Kieffer, 1911) | 1 | 477 (477–477) |
| *Stempellinella minor* (Edwards, 1929) | 1 | 725 (725–725) |
| *Tanytarsus bathophilus* (Kieffer, 1911) | 3 | 740 (725–787) |
| *Tanytarsus brundini* (Lindeberg, 1963) | 1 | 477 (477–477) |
| *Tanytarsus buchonius* (Reiss & Fittkau, 1971) | 15 | 500 (98–801) |
| *Tanytarsus chinyensis* (Goetghebuer, 1934) | 9 | 293 (111–772) |
| *Tanytarsus curticornis* (Kieffer, 1911) | 5 | 607 (110–787) |
| *Tanytarsus ejuncidus* (Walker, 1856) | 1 | 125 (125–125) |
| *Tanytarsus fimbriatus* (Reiss & Fittkau, 1971) | 3 | 732 (477–833) |
| *Tanytarsus gibbosiceps* (Kieffer, 1922) | 5 | 125 (109–183) |
| *Tanytarsus glabrescens* (Edwards, 1929) | 10 | 563 (125–1847) |
| *Tanytarsus gregarius* (Kieffer, 1909) | 8 | 580 (164–1306) |
| *Tanytarsus heusdensis* (Goetghebuer, 1923) | 1 | 787 (787–787) |
| *Tanytarsus lestagei* agg. | 10 | 679 (111–801) |
| *Tanytarsus medius* (Reiss & Fittkau, 1971) | 6 | 536 (111–787) |
| *Tanytarsus mendax* (Kieffer, 1925) | 1 | 824 (824–824) |
| *Tanytarsus nigricollis* (Goetghebuer, 1939) | 5 | 467 (139–619) |
| *Tanytarsus pallidicornis* (Walker, 1856) | 2 | 607 (505–709) |
| *Tanytarsus signatus* (van der Wulp, 1859) | 1 | 787 (787–787) |
| *Tanytarsus* cf. *smolandicus* (Brundin, 1947) | 1 | 725 (725–725) |
| *Tanytarsus sylvaticus* (van der Wulp, 1859) | 1 | 477 (477–477) |
| *Tanytarsus usmaensis* (Pagast, 1931) | 4 | 463 (111–621) |
| *Tanytarsus* Pe 4 (Langton, 1991) | 7 | 754 (619–920) |
| *Zavrelia pentatoma* (Kieffer & Bause, 1913) | 2 | 827 (740–913) |
